# Allergy in the Elderly: A Broad Clinical Spectrum Beyond Atopy

**DOI:** 10.3390/medicina62061010

**Published:** 2026-05-23

**Authors:** Fikriye Kalkan, Begum Gorgulu Akin, Sarpcan Maden, Makbule Seda Bayrak Durmaz, Betul Ozdel Ozturk, Orhun Efe, Sadan Soyyigit

**Affiliations:** 1 Allergy and Clinical Immunology, Ankara Bilkent City Hospital, Ankara 06800, Turkey; sarpcanmaden@gmail.com (S.M.); dr.seda_bayrak@hotmail.com (M.S.B.D.); betulozdel84@gmail.com (B.O.O.); dr.orhunefe@gmail.com (O.E.); 2 Division of Allergy and Clinical Immunology, Department of Chest Diseases, School of Medicine, Ankara Yildirim Beyazit University, Ankara 06800, Turkey; drbegumgorgulu@gmail.com (B.G.A.); sadansoyyigit@gmail.com (S.S.)

**Keywords:** elderly, allergy, drugs

## Abstract

*Background:* This study aimed to evaluate referral reasons, distribution of allergic diseases, atopic status, and comorbidity associations among patients aged 65 years and older presenting to a tertiary allergy clinic. *Methods:* This retrospective study included all geriatric patients (≥65 years) who attended the Immunology and Allergy outpatient clinic at Ankara Bilkent City Hospital between January 2024 and December 2025. Demographic characteristics, comorbidities, referral complaints, and allergic diagnoses were recorded. Allergen sensitization was assessed using skin tests and/or allergen-specific IgE measurements. *Results:* A total of 1302 geriatric patients were included (mean age 70.9 years; 59.8% female). At least one comorbidity was present in 62.6% of patients, with hypertension being the most common(39.4%). The leading referral complaints were rhinorrhoea/sneezing (22.8%), pruritus (19.1%), drug allergy/adverse drug reactions (14.8%), and chronic urticaria (10.9%). The most common diagnoses were rhinitis (63.2% non-allergic), non-allergic pruritus, drug allergy, and chronic urticaria. Among inhalant allergens, pollen sensitivity (42.2%) was most frequent, followed by house dust mite (32.5%). The most frequently implicated drug groups were antibiotics (42.4%) and analgesics (21.7%). Chronic urticaria and ACE inhibitor-associated angioedema showed significant gender differences: 68.6% female (*p* = 0.001) and 66.7% male (*p* = 0.008), respectively. Patients with asthma, rhinitis, or angioedema frequently had comorbid conditions (91.1%, 55.8%, and 83.7%, *p* = 0.001, *p* = 0.013, and *p* = 0.001, respectively). *Conclusions:* Allergy clinic presentations in elderly patients reflect a broad clinical spectrum, including non-allergic conditions, frequent drug-related reactions in elderly patients with multiple comorbidities, and age-related immunological changes alongside atopic diseases. A comprehensive, individualized diagnostic approach is essential when evaluating allergic complaints in the geriatric population.

## 1. Introduction

Life expectancy and the elderly population are increasing globally. Concurrently, the incidence of allergic diseases among the elderly is rising. This increase partly reflects improved identification of the pathogenesis underlying previously unexplained symptoms. Additionally, individuals who were allergic two decades ago now constitute the elderly demographic. Environmental pollution and age-related structural changes further increase susceptibility to conditions such as allergic asthma and dermatitis. Lymphocyte counts decline during early life, stabilize at low levels in young adulthood, and drop further after age 60. Reduced T cell function, impaired antibody production, and altered mast cell activity influence allergen responses and inflammation in this population [1,2].

The thymus, an important organ for the immune system, shrinks rapidly from adolescence onwards. The decrease in functional volume causes reduced naive T cell proliferation and increased memory T cell proliferation. Despite the thymus shrinking, the total T cell pool remains constant. This is due to the proliferation of memory T cells [3]. The shift towards Th2 leads to relative dominance of the type 2 immune response. As a result, susceptibility to diseases such as asthma, rhinitis, and atopic dermatitis increases [4,5].

In recent years, the increase in asthma prevalence has been most pronounced among the elderly. Although asthma can begin at any age, asthma that develops in old age is generally non-atopic, more severe, and persistent compared to asthma that begins at a young age [6]. Adverse drug reactions occur more frequently in elderly patients than in younger populations, contributing to significant morbidity and mortality [7].

In dermatological allergies, skin dryness promotes the development of allergic contact dermatitis and atopic dermatitis. Additionally, mucous membrane aging and reduced IgA secretion predispose elderly individuals to other allergic conditions. Polypharmacy in this population is also associated with increased drug reactions [8,9].

In this context, managing allergic diseases in elderly individuals should not be limited to symptom control. It should also address the functional causes of these diseases, their relationship with comorbidities, and appropriate management. Customized allergy management in elderly individuals requires a multidisciplinary approach across both diagnosis and treatment.

This study aimed to elucidate the reasons for referral of patients aged 65 and older to the allergy clinics at Ankara Bilkent City Hospital. Additionally, it sought to identify the allergic diseases and atopic status present in this population following clinical examination. The primary objective was to quantify referral frequencies for specific complaints, categorized as respiratory symptoms (e.g., rhinitis, cough, asthma), cutaneous symptoms (e.g., pruritus, urticaria, eczema), and suspected drug reactions. Secondary objectives included determining the prevalence and atopic status of confirmed allergic diseases based on clinical and laboratory data, and examining associations between these diagnoses and comorbidities. These aims were established to align with subsequent statistical analyses.

## 2. Materials and Methods

### 2.1. Study Design

This retrospective study included all patients aged 65 and older who attended the Immunology and Allergy outpatient clinic at Ankara Bilkent City Hospital between January 2024 and December 2024. Consecutive patients meeting the eligibility criteria during the study period were included in the analysis. The two-year period was chosen to ensure adequate patient volume and to account for potential seasonal variations in allergic diseases. Demographic characteristics, comorbidities, reasons for outpatient visits, and allergic diagnoses established during clinical evaluations were analyzed. Ethical approval was obtained from the Ankara Bilkent City Hospital Local Ethics Committee (Approval No: TABED 2-25-1334). The study adhered to the principles of the Declaration of Helsinki.

### 2.2. Data Collection

All patients aged 65 and older presenting to the immunology and allergy outpatient clinic were included, except those with incomplete clinical records, insufficient diagnostic evaluation, or missing key clinical data were excluded from the study. Demographic, clinical, and laboratory data were extracted from the hospital’s electronic medical records. These data encompassed age, sex, comorbidities, reasons for outpatient visits, physical examination findings, and results of laboratory and allergy tests. Evaluations included allergen-specific IgE levels, serum tryptase, skin prick tests (SPTs), patch tests, and provocation tests when indicated. Serum-specific IgE testing was performed selectively according to clinical indications and physician evaluation and was not systematically available for all patients.

Allergen sensitivity was assessed using in vivo skin prick tests and/or in vitro measurements of allergen-specific IgE levels. Allergen-specific IgE was quantified using the Immulite 2000 system (Siemens Healthcare Diagnostics, Tarrytown, NY, USA), which has an analytical sensitivity of 0.10 kU/L. Values above 0.35 kU/L were considered positive. SPTs employed commercial allergen extracts (Lofarma, Milan, Italy) and included inhalant allergens such as Artemisia vulgaris, Aspergillus fumigatus, Pinus sylvestris, cat epithelium, Parietaria judaica, Betula mix, oak mix, Secale cereale, Phleum pratense, Olea europaea, Blattella germanica, Plantago lanceolata, dog epithelium, Lamb’s quarters, Lolium perenne, Cupressus arizonica, Populus nigra, Alternaria alternata, Penicillium chrysogenum, wild grasses, Cladosporium, honeybee, wasp and food allergens. Histamine (10 mg/mL) served as the positive control, and 0.9% NaCl as the negative control. İn cases where both honeybee and wasp are positive, component-resolved diagnostics were performed to evaluate IgE antibodies against individual allergen components (e.g., Api m 1, Api m 2 for honeybee; Ves v 1, Ves v 5 for wasp), rather than whole extracts. This approach helps differentiate cross-reactivity from true double sensitization [10].

Patch testing utilized the European Standard Series (ESS), comprising 30 allergens (Chemotechnique Diagnostics, Vellinge, Sweden). Allergens were prepared with Van der Bend Chambers at recommended concentrations. Tests were applied to the upper back, with patches removed at 48 h for the initial reading and final readings conducted at 96 h [11,12].

Allergic diagnoses were established according to clinical history, physical examination findings, laboratory evaluation, and allergy testing results in accordance with current guideline-based clinical practice [13].

### 2.3. Statistical Analysis

Data were analyzed using SPSS version 25.0. Continuous variables are presented as mean ± standard deviation, and categorical variables as counts and percentages. Group comparisons employed the Chi-square test or Fisher’s exact test for categorical variables, and Student’s *t*-test or Mann–Whitney U test for continuous variables. Logistic regression analyses were performed for outcomes with sufficient event frequency and clinical relevance. Statistical significance was set at *p* < 0.05.

## 3. Results

The study included 1302 geriatric patients with a mean age of 70.9 years (range: 65–93 years). Among them, 523 (40.2%) were male and 778 (59.8%) were female. A total of 814 patients (62.6%) had at least one comorbid condition, while 487 (37.4%) had none. Hypertension was the most prevalent comorbidity (39.4%). The most common symptoms leading to allergy clinic visits were rhinorrhea and sneezing (*n* = 326, 22.8%) and pruritus (*n* = 274, 19.1%). In addition, suspected drug allergy or adverse drug reactions (*n* = 212, 14.8%) and chronic urticaria (*n* = 156, 10.9%) were among the most frequent referral diagnoses. Demographic characteristics and reasons for referral are detailed in Table 1. Combined symptom presentations were categorized separately and were not duplicated within isolated symptom groups.

Table 2 presents the distribution of diagnoses by gender among geriatric patients based on clinical examination. The most common diagnoses were rhinitis (*n* = 326), non-allergic pruritus (*n* = 255), drug allergy (*n* = 212), and chronic urticaria (*n* = 156). Non-allergic rhinitis was among the most common reasons for referral to the allergy clinic in the elderly population. Symptoms such as rhinorrhea and sneezing accounted for nearly 25% of referrals, exceeding complaints typically associated with classical allergic conditions. Chronic urticaria occurred significantly more frequently in women (*p* = 0.001), particularly within the chronic spontaneous urticaria subgroup (*p* = 0.009). Angioedema related to ACE inhibitors was significantly more prevalent in men (*p* = 0.008). Drug allergy was generally more common in women, with adverse drug reactions showing a significant difference (*p* = 0.016). Hymenoptera venom allergy was more frequent in men overall (*p* = 0.010) and specifically in the Vespula venom allergy subgroup (*p* = 0.014). Nasal obstruction-related diseases were more common in men (*p* = 0.011), primarily due to the Non-steroidal anti-inflammatory drug Exacerbated Respiratory Disease (NERD)subgroup (*p* = 0.019). Cough was more frequent in women (*p* = 0.001), especially non-atopic cough (*p* = 0.001). No significant gender differences were observed for eczema-dermatitis, food allergy, idiopathic anaphylaxis, or non-allergic pruritus (Table 2).

**Figure 1:**
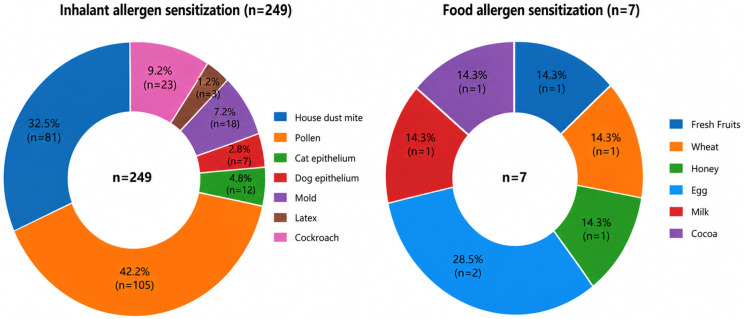
Distribution of inhalant and food allergen sensitization among sensitized patients.

The aeroallergen and food allergen sensitization profiles are summarized in Figure 1. Among inhalant allergens, sensitization was most common to pollens (42.2%), followed by house dust mites (32.5%). Sensitization to food allergens was infrequent and primarily involved eggs, milk, wheat, honey, cocoa, and certain fruits (Figure 1).

The most frequently implicated drug group was antibiotics (*n* = 86, 42.4%), followed by analgesics (*n* = 44, 21.7%). Among antibiotics, beta-lactams (*n* = 59, 28.6%) and quinolones (*n* = 13, 6.3%) were the most prevalent. Among analgesics, non-steroidal anti-inflammatory drugs (NSAID) accounted for the majority of reactions (*n* = 40, 20.0%). Platinum-based agents were the most common chemotherapeutic drugs involved (*n* = 12, 5.9%). Type IV hypersensitivity reactions most frequently presented as maculopapular eruptions associated with beta-lactam antibiotics (*n* = 3, 1.5%). Additionally, severe cutaneous drug reactions such as Stevens–Johnson syndrome linked to ciprofloxacin and fixed drug eruptions related to naproxen sodium were observed. Table 3 presents the distribution of drug groups that cause hypersensitivity reactions.

Table 4 presents the distribution of allergic diseases according to comorbidities. Significant associations were identified between asthma (*p* = 0.001), rhinitis (*p* = 0.013), and angioedema (*p* = 0.001) and the presence of comorbid conditions. No significant associations were observed for chronic urticaria, cough, nasal obstruction, or drug allergy with comorbidities (Table 4).

Table 5 illustrates that the presence of comorbidities was the strongest independent predictor for both asthma and angioedema in the binary logistic regression analyses. Individuals with comorbidities had significantly higher odds of asthma (OR = 12.133, 95% CI: 4.879–30.173, *p* < 0.001) and angioedema (OR = 3.120, 95% CI: 1.444–6.738, *p* = 0.004). In the asthma model, patients aged 75 years and older showed significantly lower odds of asthma compared to the 65–69 age group (OR = 0.525, 95% CI: 0.291–0.944, *p* = 0.032), whereas age was not significantly associated with angioedema risk. Male sex was identified as an independent predictor of angioedema (OR = 2.044, 95% CI: 1.145–3.651, *p* = 0.016), while sex was not significantly associated with asthma presence. Overall, the asthma model demonstrated a better discriminative performance than the angioedema model (AUC: 0.719 vs. 0.673).

## 4. Discussion

The growing elderly population worldwide necessitates a detailed evaluation of diseases and clinical presentations characteristic of this age group. Identifying presenting complaints and diagnostic distributions among geriatric patients attending allergy clinics is essential to delineate relevant clinical features. This study examined reasons for presentation and allergic and non-allergic diagnoses in patients aged 65 and older. The findings are expected to enhance understanding of the manifestations of allergic disease in the elderly and to inform diagnostic approaches for this population.

Prevalence estimates suggest that rhinitis symptoms affect up to 32% of elderly individuals [14]. Mucosal dryness, crusting, cough, and decreased olfactory function may be more pronounced in older adults. Age-related structural and functional changes, e.g., decreased nasal cartilage support, mucosal atrophy, decreased hydration, impaired mucociliary clearance, and loss of smell, may affect the onset and severity of symptoms [5,15,16,17]. These changes likely contribute to the observed association between rhinitis and comorbidities in the elderly. Conditions, including chronic respiratory and cardiovascular diseases, as well as polypharmacy, may facilitate rhinitis symptoms and exacerbate clinical burden. Thus, the significant association between rhinitis and comorbidities in this study indicates multifactorial influences on rhinitis in elderly individuals.

The literature indicates that the prevalence of allergic rhinitis is lower in elderly populations than in young adults [18,19,20]. For instance, the German ESTHER study, involving 9949 individuals aged 50–75, reported an allergic rhinitis prevalence of 8.3% [20]. Another geriatric study with 7124 patients found seasonal allergic rhinitis prevalence of 12.6% and perennial allergic rhinitis prevalence of 17.1%, with pollens and house dust mites as the most frequent sensitizers [21]. Similarly, a separate study reported a 3.8% prevalence of allergic rhinitis in elderly patients, with pollen sensitivity predominating [22]. Some studies, however, suggest house dust mite sensitivity is more prevalent in elderly individuals [19,20,21,22,23]. In the present study, rhinitis was a prominent complaint among elderly outpatients. Among patients with rhinitis symptoms, 63.2% had non-allergic rhinitis, while the prevalence of allergic rhinitis was 9.2% in the entire elderly cohort. The most frequent sensitivities in patients with allergic rhinitis were to pollens, followed by house dust mites. These results indicate that non-allergic rhinitis predominates in the elderly, although allergic rhinitis remains common, with inhalant allergen sensitivities consistent with the existing literature. Additionally, consistent with this study, multiple reports indicate that nasal polyps are more frequent in men [24,25]. Although the classical atopic march is primarily described in pediatric populations, some findings in our cohort may reflect persistent atopic phenotypes extending into older age. However, due to the retrospective cross-sectional design, definitive conclusions regarding the atopic march cannot be established. The coexistence of allergic and non-allergic conditions in elderly patients may reflect the complex interaction between immunosenescence, chronic systemic inflammation, multimorbidity, and cumulative environmental or medication exposure. In addition, age-related structural and functional alterations may modify the clinical expression of classical allergic diseases and contribute to overlapping symptom patterns in geriatric populations [1]. The coexistence of allergic and non-allergic conditions in elderly patients may reflect the complex interaction between immunosenescence, chronic systemic inflammation, multimorbidity, and cumulative environmental or medication exposure [26,27,28].

Hymenoptera venom allergy is reported to be more prevalent in men [29]. Consistent with this, venom allergy was more frequent among men in the present series.

Adverse drug reactions are more frequent in elderly patients than in younger populations, resulting in significant morbidity and mortality. Advanced age is an independent risk factor for hospitalization due to drug-related reactions [7,30]. Evidence suggests that aging increases susceptibility to both immediate and delayed hypersensitivity reactions and drug allergies [31,32]. Beta-lactam antibiotics and NSAIDs are the most common agents implicated in drug hypersensitivity among elderly patients [33,34]. Consistent with these findings, the present study observed the highest susceptibility rates to beta-lactam antibiotics and NSAIDs. The relatively high frequency of drug hypersensitivity reactions may also be associated with repeated drug exposure, polypharmacy, and cumulative sensitization, which are common in elderly patients with multiple comorbidities. Given that many cases were referrals to allergy clinics, Type I hypersensitivity reactions predominated. Delayed-type reactions are typically presented as maculopapular eruptions. Regarding chemotherapeutic agents, Type I hypersensitivity reactions were more frequent, especially with taxanes and platinum-based drugs, consistent with the literature [35,36].

In clinical practice, asthma onset in the elderly is frequently misdiagnosed as chronic obstructive pulmonary disease (COPD), resulting in suboptimal treatment. Diagnostic challenges arise from the heterogeneous clinical and functional presentation of geriatric asthma, partial loss of airway obstruction reversibility, and a less prominent allergic component [37]. Female sex, comorbidities, infections, and low socioeconomic status have been identified as factors increasing disease burden in elderly asthmatics [38]. Aging-related structural lung changes combined with airway remodeling in asthma may exacerbate disease severity and impair respiratory function. The Global Initiative for Asthma (GINA) report indicates that asthma prevalence in the general population ranges from 5 to 10%, with 3–5% classified as severe asthma [39,40]. In this study, 6.9% (*n* = 90) of patients had asthma, and 22.2% (*n* = 20) had severe asthma. The elevated rate of severe asthma may reflect its increased severity and persistence in elderly patients, as well as continuation of severe asthma phenotypes diagnosed earlier in adulthood. Severe asthma is therefore more common and persistent in the elderly, necessitating careful recognition and individualized management. Biological therapies have been reported to reduce annual exacerbations, are well tolerated, and yield clinical outcomes comparable to those of younger adults [41,42,43]. Collectively, these data underscore the need to consider respiratory, structural, immunological, and clinical factors related to aging in the diagnosis and management of asthma in elderly patients.

In the multivariable analysis, the presence of comorbidities emerged as the strongest independent predictor for both asthma and angioedema. This finding may reflect the complex clinical burden and chronic inflammatory background commonly observed in older adults with allergic diseases. Male sex was independently associated with angioedema, which may indicate potential sex-related differences in susceptibility or healthcare utilization patterns. In contrast, individuals aged 75 years and older demonstrated lower odds of asthma compared to the younger elderly subgroup, possibly reflecting survivor bias, underdiagnosis, or age-related changes in allergic inflammatory responses. Nevertheless, these findings should be interpreted cautiously due to the retrospective design of the study.

Chronic pruritus is increasingly recognized as a significant health issue in elderly individuals. Its etiology is predominantly multifactorial, involving physiological changes in aging skin, impaired barrier function, immune alterations, neurological factors, and psychological influences. Additionally, the higher prevalence of comorbidities and polypharmacy in this population elevates the risk of drug-induced pruritus or exacerbation of existing symptoms. The literature emphasizes that pruritus in elderly patients is often non-allergic and is commonly associated with dry skin, systemic diseases, and other age-related conditions [8,44]. In this study, 19.6% (*n* = 255) of elderly patients presented with pruritus, primarily attributed to dry skin and comorbid disease-related itching, consistent with prior reports. Nevertheless, the severity of pruritus prompted many patients to seek evaluation for potential allergic causes. Urticaria in elderly patients is frequently linked to comorbidities and malignancies and is more prevalent in women [45]. Correspondingly, chronic spontaneous urticaria was more common among female patients and those with additional diseases in this cohort.

Community-based data indicate that angioedema etiology is predominantly idiopathic (40–50%) and ACE inhibitor (ACEI)-associated (25–35%), with hereditary angioedema occurring less frequently (1–5%) [46]. While mast cell-mediated urticaria and angioedema persist in the elderly, bradykinin-mediated angioedema is increasingly prominent, likely due to higher ACEI usage in this population. Acquired C1 inhibitor deficiency, a bradykinin-mediated subtype, is most common in advanced age. Drug-induced angioedema from agents such as ACEIs, aspirin, and nonsteroidal anti-inflammatory drugs is also more prevalent among elderly individuals [47]. The present study corroborates these findings, reporting similar rates of idiopathic angioedema (51%, *n* = 25) and ACEI-associated angioedema (42.9%, *n* = 21), with a significant association between angioedema and comorbidities. These results suggest that ACEI-associated angioedema may impose a greater clinical burden in elderly patients, potentially secondary to comorbid conditions. Additionally, the significantly higher incidence of ACEI-associated angioedema in male patients aligns with existing literature [48].

Cough is another frequent reason for visits to the allergy clinic. Chronic cough affects approximately 10% of the general adult population. In elderly individuals, cough etiology is more complex due to age-related declines in respiratory muscle strength and higher prevalence of comorbidities such as gastroesophageal reflux, asthma, postnasal drip, and ACE inhibitor use, which are leading causes in this age group [49,50]. In this study, non-atopic causes predominated among patients presenting with cough, suggesting that cough in elderly patients often arises from respiratory, gastrointestinal, or drug-related factors rather than allergic mechanisms. Additionally, cough-related clinic visits were more frequent among female patients, consistent with the literature, which attributes this to increased cough reflex sensitivity in women [51].

Despite these insights, several important questions remain unresolved and warrant further investigation. For instance, how do patterns of allergic and non-allergic presentations vary across different regions, healthcare settings, and populations? What factors contribute to variations in allergic disease expression, severity, and management outcomes among elderly individuals with diverse comorbid profiles? Future multicenter, collaborative studies are needed to address these questions and to develop evidence-based, standardized strategies for evaluating and managing allergic diseases in the aging population. Such research would enhance the foundation for personalized and effective care in this expanding demographic.

Compared with younger populations reported in the literature, elderly patients appear to demonstrate a higher frequency of non-allergic presentations, drug-related reactions, comorbidity-associated symptoms, and chronic pruritus, whereas classical IgE-mediated allergic diseases may be relatively less prominent. These findings support the concept that allergic disease expression changes with aging and may differ substantially from that of younger adults [26,52].

The primary limitations of this study include its retrospective single-center design and the absence of a control group. It was not possible to fully isolate the effects of comorbidities, frequent medication exposure, and age-related physiological changes on certain clinical complaints. In addition, the lack of a younger comparison group limited direct evaluation of age-specific differences and may reduce the external validity of the findings. Because the study population was derived from a tertiary referral allergy clinic, referral bias may have influenced the observed disease distribution and may not fully represent the general geriatric population. Furthermore, detailed information regarding prior sensitization history or childhood atopic disease was not consistently available due to the retrospective nature of the study.

## 5. Conclusions

Collectively, these findings suggest that allergy clinic visits among elderly patients encompass a broad clinical spectrum, including allergic diseases, age-related immunological alterations, comorbidities, and structural and functional changes. Therefore, evaluation of allergic complaints in elderly patients should also consider non-allergic causes, drug reactions associated with frequent medication exposure and comorbid diseases, in addition to atopic mechanisms.

This study provides descriptive data regarding the causes and diagnostic distribution of allergy clinic visits in a geriatric population evaluated at a tertiary referral center. Although allergic diseases are often associated with younger populations, they may also represent an important source of morbidity in older adults. Accordingly, a careful and individualized diagnostic approach may be beneficial when evaluating allergic conditions in elderly patients. However, the retrospective single-center design limits the generalizability of the findings, and further multicenter studies are warranted.

## Figures and Tables

**Table 1:** Demographic findings of geriatric patients and reasons for referring to the allergy clinic.

Variable	Total (n = 1301), n (%)
**Demographic characteristics**	
Age, years, median (range)	70.9 (65–93)
Female sex	778 (59.8)
**Comorbidities**	
No comorbid condition	487 (37.4)
Hypertension	513 (39.4)
Diabetes mellitus	174 (13.4)
Asthma	138 (10.6)
Cardiovascular disease	158 (12.1)
Thyroid disease	96 (7.1)
Renal and urinary system disease	40 (3.1)
Gastrointestinal disease	18 (1.4)
Respiratory system disease	50 (3.8)
Malignancy	49 (3.8)
Rheumatologic disease	34 (2.6)
Neuropsychiatric disease	25 (1.9)
**Presenting symptoms**	
Rhinorrhea and sneezing	326 (22.8)
Pruritus	274 (19.1)
Cough	87 (6.1)
Dyspnea	49 (3.4)
Dyspnea with rhinorrhea	34 (2.4)
Nasal congestion	15 (1.0)
Rash	13 (1.0)
Postnasal drip	6 (0.4)
Abdominal bloating	3 (0.2)
**Clinical indications for referral**	
Drug hypersensitivity reactions and/or adverse drug reactions	212 (14.8)
Chronic urticaria	156 (10.9)
Angioedema	50 (3.5)
Eczema/dermatitis	45 (3.1)
Suspected food allergy	37 (2.6)
Hymenoptera venom reaction	30 (2.1)
Recurrent infections and/or chronic diarrhea history	27 (1.9)
Metal-related contact dermatitis	17 (1.2)
Nasal polyposis	12 (0.8)
Evaluation of elevated total IgE levels	12 (0.8)
Severe asthma	11 (0.8)
History of acute urticaria	5 (0.4)
History of idiopathic anaphylaxis	5 (0.4)
Evaluation of eosinophilia	3 (0.2)
Hereditary angioedema follow-up	3 (0.2)

**Abbreviations:** IgE: Immunoglobulin E. **Footnote:** Patients categorized under combined symptom groups (e.g., dyspnea with rhinorrhea) were not additionally included in isolated symptom categories.

**Table 2:** Gender-based distribution of diagnoses according to examination findings in geriatric patients.

Disorders. n (%)	Total	Male	Female	*p* Value
Rhinitis	326	113	195	0.308
Allergic	120	57 (47.5)	63 (52.5)	0.539
Non-allergic	206	74 (35.9)	132 (64.1)	0.172
Bronchial asthma	90	29	61	0.586
Allergic	29	8 (27.6)	21 (72.4)	0.161
Non-allergic	41	14 (34.1)	27 (65.9)	0.422
Severe asthma—omalizumab	10	2 (20)	8 (80)	0.332
Severe asthma—mepolizumab	8	4 (50)	4 (50)	NA
Severe asthma—benralizumab	2	1 (50)	1 (50)	NA
Acute urticaria	5	2 (40)	3 (60)	NA
Chronic urticaria	156	49	107	**0.001**
Spontaneous	112	35 (31.3)	77 (68.8)	**0.009**
Inducible	4	2 (50)	2 (50)	NA
Urticaria with angioedema	25	8 (32)	17 (68)	0.517
Using Omalizumab	15	4 (26.7)	11 (73.3)	0.178
Eczema-dermatitis	61	31	30	0.508
Contact dermatitis	37	17 (45.9)	20 (54.1)	0.753
Non-contact dermatitis	24	14 (58.3)	10 (41.7)	0.082
Angioedema	49	28	21	0.392
Idiopathic	25	14 (56)	11 (44)	0.113
ACE inhibitor—induced angioedema	21	14 (66.7)	7 (33.3)	**0.008**
Hereditary angioedema	3	0	3 (100)	0.251
Drug hypersensitivity and adverse drug reactions	212	83	129	0.642
Type-1 hypersensitivity reactions	189	70 (37)	119 (63)	0.199
Type-4 hypersensitivity reactions	11	4 (36.3)	7 (63.7)	0.715
Infusion reactions	3	2 (66.7)	1 (33.3)	NA
Other adverse drug reactions	9	7 (77.8)	2 (22.2)	**0.016**
Food allergy	7	4 (57.1)	3 (42.9)	NA
Hymenoptera venom allergy	30	21	9	**0.010**
Apis	12	8 (66.7)	4 (33.3)	0.149
Vespula	15	11 (73.3)	4 (26.7)	**0.014**
Vespula + Apis	3	2 (66.7)	1 (33.3)	NA
Idiopathic Anaphylaxis	5	1 (20)	4 (80)	0.367
Nasal obstruction	16	13	3	**0.011**
Nasal polyps	4	3 (75)	1 (25)	0.617
NERD	7	6 (85.7)	1 (14.3)	**0.019**
Nasal polyps treated with mepolizumab	3	2 (66.7)	1 (33.3)	NA
Nasal polyps treated with benralizumab	1	1 (100)	0	NA
EGPA	1	1	0	NA
Cough	79	15	64	**0.001**
Atopic	12	6 (50)	6 (50)	NA
Nonatopic	57	5 (8.8)	52 (91.2)	**0.001**
ACE inhibitors associated	10	4 (40)	6 (60)	0.754
Non-allergic pruritis	255	122 (47.8)	133 (52.2)	0.531

**Abbreviations:** NSAID: Non -Steroidal Anti-Inflammatory Drug, ACE: Angiotensin-converting enzyme, NERD: NSAID exacerbated respiratory disease, EGPA: eosinophilic granulomatosis with polyangiitis.

**Table 3:** Distribution of drug hypersensitivity reactions.

Variables	N (%)
**Type-1 hypersensitivity reactions**	**189 (89.1)**
Analgesics	44 (20.7)
NSAID	40 (18.8)
Paracetamol	4 (1.9)
Antibiotics	86 (40.5)
Beta- Lactam	59 (27.8)
Macrolide	4 (1.9)
Quinolone	13 (6.1)
Trimethoprim Sulfamethoxazole	3 (1.4)
Ornidazole—Metronidazole	4 (1.9)
Nitrofurantoin	2 (0.9)
Tetracycline	1 (0.4)
Cardiovascular drugs	4 (1.9)
Proton pump inhibitors	8 (3.7)
Local Anesthetics	5 (2.3)
General Anesthetic	5 (2.3)
Contrast Agent	12 (5.6)
Chemotherapeutics	17 (8)
Platinum Group	10 (4.7)
Taxanes Group	6 (2.8)
Other Chemotherapeutics	1 (0.4)
Other drugs	8 (3.7)
Allopurinol	1 (0.4)
Antiacidosis	1 (0.4)
Atorvastatin	1 (0.4)
B-vitamin complex	2 (0.9)
Colchicine	2 (0.9)
Hyoscine butylbromide	1 (0.4)
**Type-4 hypersensitivity reactions**	**11 (5.3)**
Beta-Lactam Group-Related Maculopapular Eruption (MPE)	3 (1.4)
NSAIDs Related MPE	1 (0.4)
Antiepileptic (Levetiracetam) Related MPE	1 (0.4)
Taxane Related MPE	1 (0.4)
Paracetamol Related MPE	1 (0.4)
Uricolysis Related MPE	1 (0.4)
Atorvastatin-Related MPE	1 (0.4)
Ciprofloxacin-Related SJS	1 (0.4)
Naproxen Sodium Related Fixed Drug Eruption	1 (0.4)
**Infusion reactions**	**3 (1.4)**
**Other adverse drug reactions**	**9 (4.2)**

Abbreviations: MPE: Maculopapular Eruption, SJS: Steven—Johnson syndrome, NSAID: Non-Steroidal Anti-Inflammatory Drug.

**Table 4:** Distribution of allergic diseases and symptoms in elderly patients according to comorbidities.

	With Comorbidity (n, %)	Without Comorbidity (n%)	*p* Value
Chronic urticaria	101 (65.6)	53 (34.4)	0.232
Cough	58 (73.4)	21 (26.6)	0.146
Nasal obstruction	12 (75)	4 (25)	0.635
Asthma	82 (91.1)	8 (8.9)	0.001
Drug allergy	135 (63.7)	77 (36.3)	0.388
Rhinitis	182 (55.8)	144 (44.2)	0.013
Angioedema	41 (83.7)	8 (16.3)	0.001

**Table 5:** Binary logistic regression analyses for asthma presence and angioedema.

Predictors	Asthma Presence OR (95% CI)	*p*-Value	Angioedema OR (95% CI)	*p*-Value
Age: 75+ (vs. 65–69)	0.525 (0.291–0.944)	0.032	1.220 (0.594–2.505)	0.588
Age: 70–74 (vs. 65–69)	0.711 (0.426–1.187)	0.192	1.319 (0.671–2.590)	0.422
Female sex	1.273 (0.809–2.004)	0.297	Reference	—
Male sex	Reference	—	2.044 (1.145–3.651)	0.016
Presence of comorbidity	12.133 (4.879–30.173)	<0.001	3.120 (1.444–6.738)	0.004

## Data Availability

The data presented in this study are available on request from the corresponding author.
